# Quantitative evaluation of comb-structure correction methods for multispectral fibrescopic imaging

**DOI:** 10.1038/s41598-018-36088-7

**Published:** 2018-12-12

**Authors:** Dale J. Waterhouse, A. Siri Luthman, Jonghee Yoon, George S. D. Gordon, Sarah E. Bohndiek

**Affiliations:** 1 Department of Physics, University of Cambridge, Cambridge, CB3 0HE UK; 2 Cancer Research UK Cambridge Institute, University of Cambridge, Cambridge, CB2 0RE UK; 3 Department of Engineering, University of Cambridge, Cambridge, CB3 0FA UK

## Abstract

Removing the comb artifact introduced by imaging fibre bundles, or ‘fibrescopes’, for example in medical endoscopy, is essential to provide high quality images to the observer. Multispectral imaging (MSI) is an emerging method that combines morphological (spatial) and chemical (spectral) information in a single data ‘cube’. When a fibrescope is coupled to a spectrally resolved detector array (SRDA) to perform MSI, comb removal is complicated by the demosaicking step required to reconstruct the multispectral data cube. To understand the potential for using SRDAs as multispectral imaging sensors in medical endoscopy, we assessed five comb correction methods with respect to five performance metrics relevant to biomedical imaging applications: processing time, resolution, smoothness, signal and the accuracy of spectral reconstruction. By assigning weights to each metric, which are determined by the particular imaging application, our results can be used to select the correction method to achieve best overall performance. In most cases, interpolation gave the best compromise between the different performance metrics when imaging using an SRDA.

## Introduction

Fibrescopes relay elements of an image via total internal reflection of light along individual fibrelets of a flexible fibre optic bundle. The term ‘fibrescope’ was first used in 1954 to describe an instrument intended for medical use in endoscopy^1^. Today, fibrescopes are used in many applications that exploit their small diameter and high flexibility, such as inspection of engines, fine diameter pipework and boilers^2^, but they are still most prominent in medical imaging, where they are known as endoscopes.

Endoscopes typically contain < 50,000 fibrelets giving a maximum resolution of <240 × 240 pixels^3^. Despite camera-on-tip endoscopes gaining popularity in many indications due to their potential for high definition imaging^4^, fibrescopes remain useful due to their superior ability to access small complex locations within the body, for example, in bronchoscopy^5,6^, cysto-nephroscopy^7^ and naso-laryngo-pharyngoscopy^8,9^ and particularly in paediatric patients where cavities are narrow. Fibrescopes also offer the ability to easily implement advanced imaging techniques, such as endomicroscopy^10^ or Raman spectroscopy^11^, when introduced into the accessory channel of camera-on-tip endoscopes^12^. For example, a commercial endomicroscope can capture high resolution *in vivo* ‘optical biopsy’ information by relaying a fluorescence signal emitted from a stain applied to the tissue (typically fluorescein) through a bundle of 30,000 fibrelets^10^.

Recent research efforts have focused on adding extra functionality to medical endoscopes to improve their ability to resolve early signs of disease, in particular, pre-cancerous lesions. One example is the integration of multispectral imaging (MSI), which enables both spatial (x, y) and spectral (wavelength, λ) information to be recorded from the tissue. MSI can extend the acquisition of spectral image data beyond the current clinically implemented endoscopic methods of autofluorescence imaging and dye-based or virtual chromoendoscopy^13^. Spectral data contains chemical information about the composition of the tissue and has been demonstrated in a wide range of potential applications in biomedical imaging^14,15^. MSI, in combination with data analysis using spectral unmixing algorithms, has been used to: visualize the vascular pattern and the oxygenation status of blood^16–22^; to improve detection of gastric^23^ and colorectal lesions;^24–26^ to identify residual tumour^27^; and to perform tissue segmentation^28,29^. MSI may also be used to improve delineation of stains applied during molecular endoscopy^30^ based on the absorption or fluorescence properties of the stain^22,31^.

Clinical implementations of spectral endoscopes are, however, currently limited by the high cost and complexity of the equipment, as well as the resulting complexity of data analysis and interpretation^32^. Several of the previously reported spectral endoscopy systems include multiple bandpass filters^25,33^, tuneable filters^24,34^, laser lines^35–37^, or detectors dedicated to separate spectral bands^35,36^; the use of multiple expensive optical components makes the systems both bulky and costly.

Recently reported spectrally resolved detector arrays (SRDAs) have the potential to overcome this problem by providing a robust compact solution for multispectral endoscopic imaging^22,38^. SRDAs integrate spectral filters directly onto the imaging detector, advancing on the conventional 2 × 2 Bayer colour filter array (CFA) super-pixel of red, green and blue filters with 3 × 3, 4 × 4 or even 5 × 5 CFA super-pixels. SRDAs therefore have an inherent trade-off between spectral and spatial resolution. Furthermore, in fibrescope applications, the number of individual fibrelets further limits the spatial resolution and introduces a comb artifact into the images due to the opaque cladding between fibrelets. It should be noted that ‘comb’ refers to an irregularly shaped and irregularly spaced artefact, whereas ‘honeycomb’ generally refers to the regular case^39^. Processing multispectral fibrescope images, for example during image co-registration or feature classification, requires careful attention to both removal of the comb artifact (“decombing”) and separation of the spectral bands (“demosaicking”), in order to maintain image quality but perhaps more importantly, spectral fidelity^40^.

Decombing methods have been extensively explored in the literature^41^. Winter *et al*. developed several Fourier-based filtering techniques and compared these to median and Gaussian filters in terms of smoothness (image variance-based) and detail preservation (resolution-based)^39^. They also developed an alternative algorithm to accurately locate and interpolate between fibrelet centres^42,43^, testing a number of interpolation strategies developed in other fields. Standard linear interpolation was found to be most suitable when low processing times are required, as in video-rate imaging^44^. Later work by Lee *et al*., Regeling *et al*. and Han *et al*. further considered different shapes and sizes of Fourier and Gaussian filters for decombing, but none of these compared their methods to the other correction strategies^40,45,46^. Additionally, none of these prior studies considered the impact of decombing on signal preservation, or quantitatively compared methods or smoothing kernel sizes. Furthermore, as they used monochrome cameras, none encountered the challenge of combined decombing and demosaicking. A recent paper by Wang *et al*. did assess an interpolation strategy similar to the method of Elter *et al*. in the context of a multispectral fibrescope with an SRDA, but only with respect to the accuracy of the spectral reconstruction and without comparison to other methods^47^.

Here, we seek to address the limitations of these previous studies by performing a thorough quantitative comparison of fibrescope decombing techniques combined with demosaicking with respect to five performance metrics defined for imaging applications: processing time, resolution, smoothness, signal and accuracy of spectral reconstruction. We test the comb correction methods of median, Gaussian and Fourier filtering, against interpolation and physical blurring in simulated monochrome images, captured monochrome images and captured multispectral images. We then evaluate the impact of demosaicking in combination with these comb corrections with respect to the same performance metrics. Finally, we create graphs from which the preferred method can be chosen based on the most important performance metrics for a given application.

## Results

**Figure 1:**
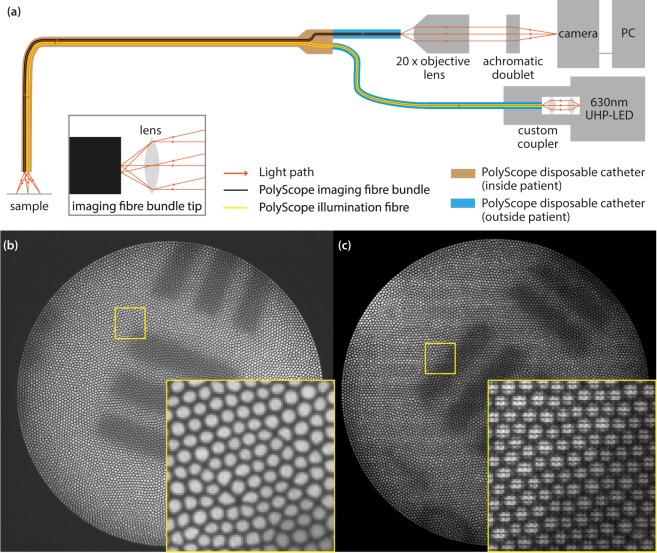
Schematic of the fibrescope and exemplar images (**a**) The system is based around the PolyScope disposable endoscope (PolyDiagnost, Germany). Illumination is provided by an ultra high-powered LED (UHP-T-LED-630, Prizmatix, Israel). Light from the PolyScope 10,000 fibre imaging bundle is focused onto either a monochrome camera (Grasshopper 3, Point Grey, Canada) or an SRDA based multispectral camera (CMS-V, SILIOS, France). Inset: Zoom of the imaging fibre tip. Rays are exaggerated for clarity. (**b**) Example comb structure from the monochrome fibrescope. Raw image of a USAF test target taken with monochrome fibrescope (cropped to ~980 × 980). Inset: Zoom of the comb structure showing irregular arrangement and shape of fibrelets. (**c**) Example comb structure and colour filter array (CFA) mosaic pattern from the multispectral fibrescope. Raw image of a USAF test target taken with multispectral fibrescope (cropped to ~980 × 950). Inset: Zoom of the comb structure showing the superimposed mosaic due to the pixel-by-pixel transmission variations of the 3 × 3 mosaic of filters deposited pixel-wise on the sensor.

We captured images experimentally by building a multispectral imaging capability into our previous endoscope design^38^ (Fig. 1a). Exemplar images captured using a monochrome camera (Fig. 1b) and multispectral SRDA (Fig. 1c) clearly demonstrate the need for comb correction and demosaicking within fibrescopic data.

### Performance of monochrome corrections to simulated monochrome images

**Figure 2:**
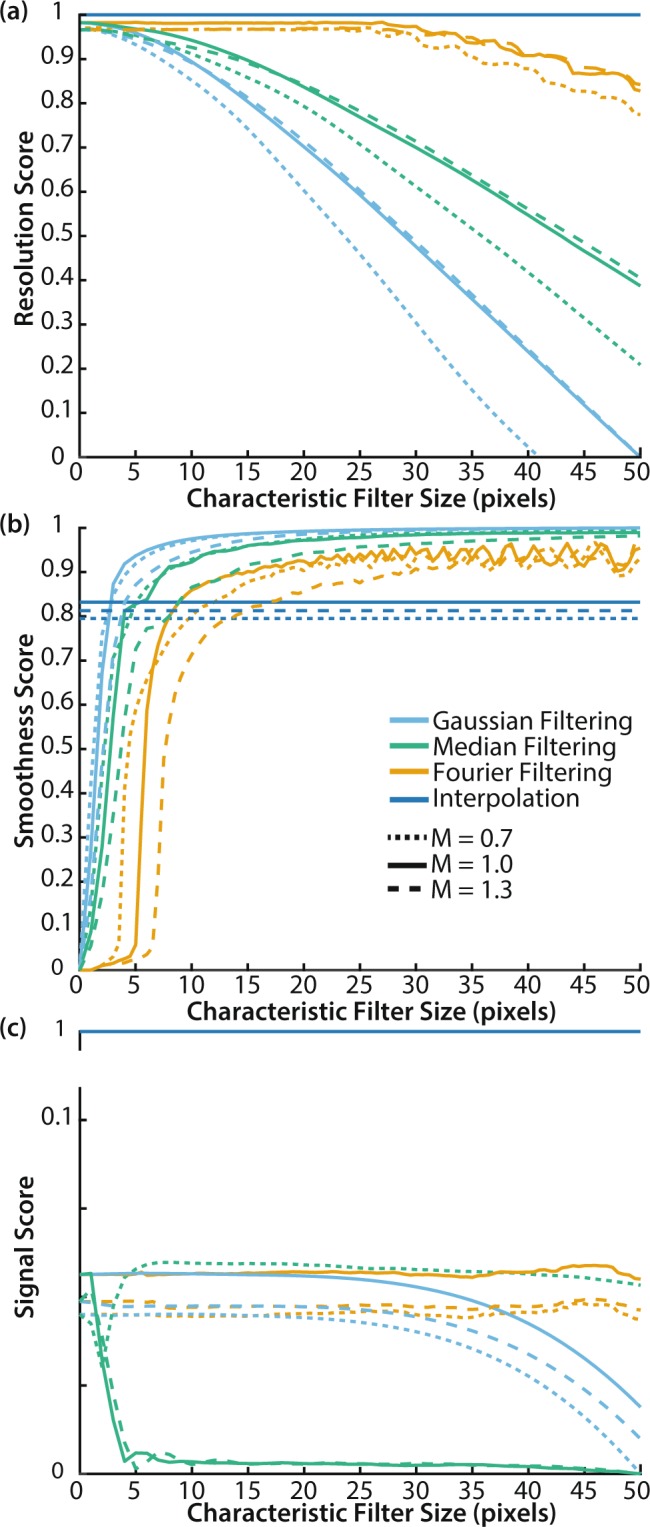
Performance scores for 4 correction methods applied to simulated monochrome images for magnifications M = 0.7, 1.0 and 1.3. (**a**) Resolution score. R_max_ = 82.3, 102, 103 pixels (648, 803, 809μm) and R_min_ = 21.4, 22.4, 23.5 pixels (169, 177, 185 μm) for M = 0.7, 1.0, 1.3 respectively. (**b**) Smoothness score. σ_max_ = 20.3, 19.9, 20.1 and σ_min_ = 0.638, 0.388, 0.346 for M = 0.7, 1.0, 1.3 respectively. (**c**) Signal score. S_max_ = 151, 156, 156 and S_min_ = 118, 117, 118 for M = 0.7, 1.0, 1.3 respectively. Gaussian filtering (sky blue), median filtering (bluish green), Fourier filtering (orange), interpolation (blue). Dotted line M = 0.7, Solid line M = 1.0, Dashed line M = 1.3. Since interpolation between irregularly spaced points is a complex spatially variant filter, the characteristic filter size is not well defined, so the score for interpolation is represented as a horizontal line in each graph.

All metrics used for evaluation of the correction methods are defined in the Methods. The scores for resolution (Fig. 2a), smoothness (Fig. 2b) and signal (Fig. 2c) as a function of the characteristic filter size for 4 decombing methods tested in simulated monochrome images were calculated for three different sized comb structures, M = 0.7, M = 1.0 and M = 1.3. These structure sizes were chosen to represent a range from 7500–30000 fibrelets within a bundle, magnified to fill a similar ~1000 pixel square sensor. For physical blurring, the correction occurs in hardware at the point of imaging. The current approach does not replicate the full imaging process, so accurate simulation of physical blurring was not possible. We do not test speed with simulated mages, since the size of the comb structure makes no difference to computation time.

**Figure 3:**
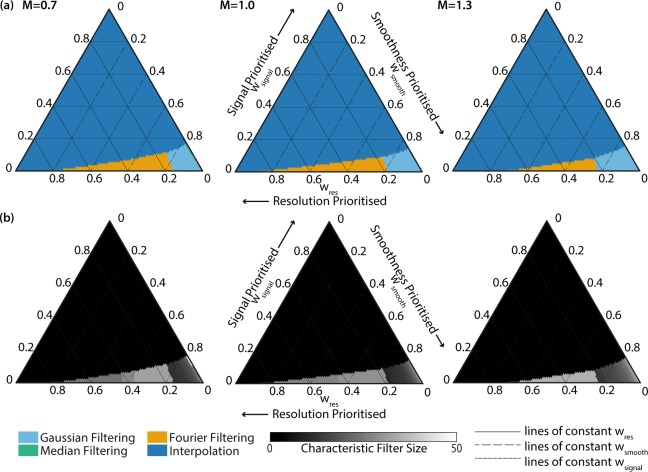
Optimum correction method based on overall performance score (OP) for weightings w_res_, w_smooth_ and w_signal_ for simulated images with magnifications M = 0.7, 1.0 and 1.3. (**a**) The correction method that gives highest OP. (**b**) The characteristic filter size used with this correction method to achieve the highest OP. Since interpolation between irregularly spaced points is a complex spatially variant filter, the characteristic filter size is not well defined, so it is represented as zero in the graphs. Speed is not included in the OP since it is possible to optimise speed independently. Gaussian filtering (sky blue), median filtering (bluish green), Fourier filtering (orange), interpolation (blue).

The size of the fibrelet has little effect on the overall trends. This is confirmed in Fig. 3, which shows the overall performance for the simulated images with different fibrelet sizes. The general shape of the optimal performance space remains constant, with the only change being a preference for median filtering in a tiny region of the performance space (around w_smooth_ = 0.85, w_signal_ = 0.15) for the smaller fibrelet dimensions (M = 0.7). This is because median filtering performs better when the filling factor, the fraction of the image filled with data (fibrelet) versus artifact (cladding), is larger, as is the case for the smaller fibrelet size. This is confirmed by cross-referencing with Fig. 3c, where the M = 0.7 median data follows a different trend to the M = 1.0 and M = 1.3 data, giving a higher signal score.

Since fibre diameter made little difference to the overall layout of performance space, we continued our investigation using captured data from our endoscope, which produced images corresponding to M = 1.0.

### Performance of monochrome corrections to captured monochrome images

**Figure 4:**
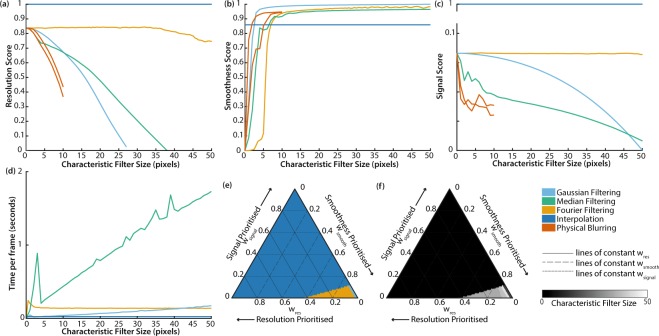
Performance scores for 5 correction methods applied to experimentally acquired monochrome images. (**a**) Resolution score. R_max_ = 500 μm and R_min_ = 228 μm. (**b**) Smoothness score. σ_max_ = 32.2 and σ_min_ = 3.85. (**c**) Signal score. S_max_ = 157 and S_min_ = 114. (**d**) Time to correct each frame. Since interpolation between irregularly spaced points is a complex spatially variant filter, the characteristic filter size is not well defined, so the score for interpolation is represented as a horizontal line in each graph. (**e**) Optimum correction method based on highest overall performance score OP for weightings w_res_, w_smooth_ and w_signal_. (**f**) The characteristic filter size used with this correction method to achieve the highest OP. Speed is not included in the OP since it is possible to optimise speed independently. Gaussian filtering (sky blue), median filtering (bluish green), Fourier filtering (orange), physical blurring (vermillion), interpolation (blue). Since interpolation between irregularly spaced points is a complex spatially variant filter, the characteristic filter size is not well defined, so it is represented as zero in the graphs.

The scores for resolution (Fig. 4a), smoothness (Fig. 4b), signal (Fig. 4c) and speed (Fig. 4d) as a function of the characteristic filter size for the 5 decombing methods tested in experimental monochrome endoscopic imaging enable direct comparisons to be made regarding their individual performance. Example images are shown in Supplementary Fig. 1.

A qualitative comparison with our simulated monochrome image corrections (Fig. 2) shows our experimental data (Fig. 4) yields similar trends. This suggests that our algorithm implementation is performing as would be expected.

The main difference is that interpolation appears to achieve an enhanced resolution score in experimental data compared to simulated data, suggesting the noise component of simulated data may have been slightly overestimated. Overestimation of the noise component would disproportionately degrade the performance of interpolation relative to the other correction methods; it is more susceptible to noise as it relies on data from a single pixel per fibrelet.

The results from our monochrome data corrections are summarised in Fig. 4e,f. For preservation of resolution and signal, interpolation clearly provides the optimal solution. If image smoothness is our only priority, Gaussian filtering is preferred. Otherwise, Fourier filtering provides a compromise between smoothness and resolution.

### Performance of multispectral corrections to captured multispectral images

**Figure 5:**
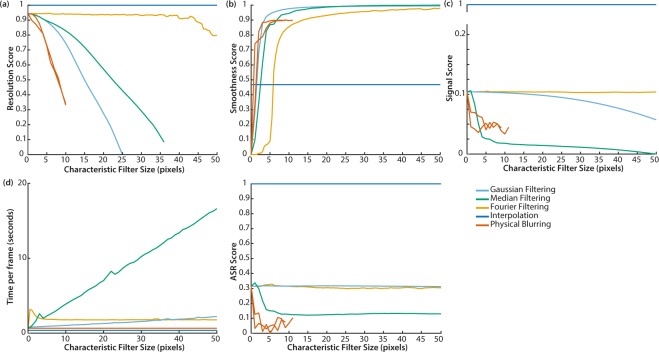
Performance scores for 5 correction methods applied to multispectral images. (**a**) Resolution score. R_max_ = 440 μm and R_min_ = 260 μm. (**b**) Smoothness score. σ_max_ = 13 and σ_min_ = 0.73. (**c**) Signal score. S_max_ = 28.1 and S_min_ = 10.6. (**d**) Speed. (**e**) Accuracy of spectral reconstruction (ASR) score. Q_max_ = 0.0138 and Q_min_ = 0.0060. Gaussian filtering (sky blue), median filtering (bluish green), Fourier filtering (orange), physical blurring (vermillion), interpolation (blue). Since interpolation between irregularly spaced points is a complex spatially variant filter, the characteristic filter size is not well defined, so the score for interpolation is represented as a horizontal line in each graph.

The scores as a function of characteristic filter size for the 5 correction methods including decombing and demosaicking as applied to multispectral images are shown in Fig. 5 for the five metrics: resolution (Fig. 5a), smoothness (Fig. 5b), signal (Fig. 5c), speed (Fig. 5d) and accuracy of spectral reconstruction (ASR) (Fig. 5e).

The ASR score is highest for interpolation, which is expected since interpolation removes the erroneous pixels from the cladding region. The other methods yield reduced ASR scores since they mix together the ‘true’ spectra from fibrelet centres and the ‘erronous’ spectra from the cladding, which is particularly evident in two cases. Firstly, in median filtering, where the sparsity of ‘true’ pixels, from the compound sampling of mosaic and comb, can result in median filters removing ‘true’ pixels over the more common ‘erroneous’ pixels. Secondly in physical blurring, where the ‘true’ and ‘erroneous’ spectra are mixed before detection.

The computation time in multispectral corrections is increased by around one order of magnitude, as expected since there are nine images to correct rather than one. The computation time of physical blurring represents demosaicking alone. If direct demosaicking were performed, resulting in 9 images each with a 9-fold reduction in total image pixels, the computation time would be significantly reduced.

**Figure 6:**
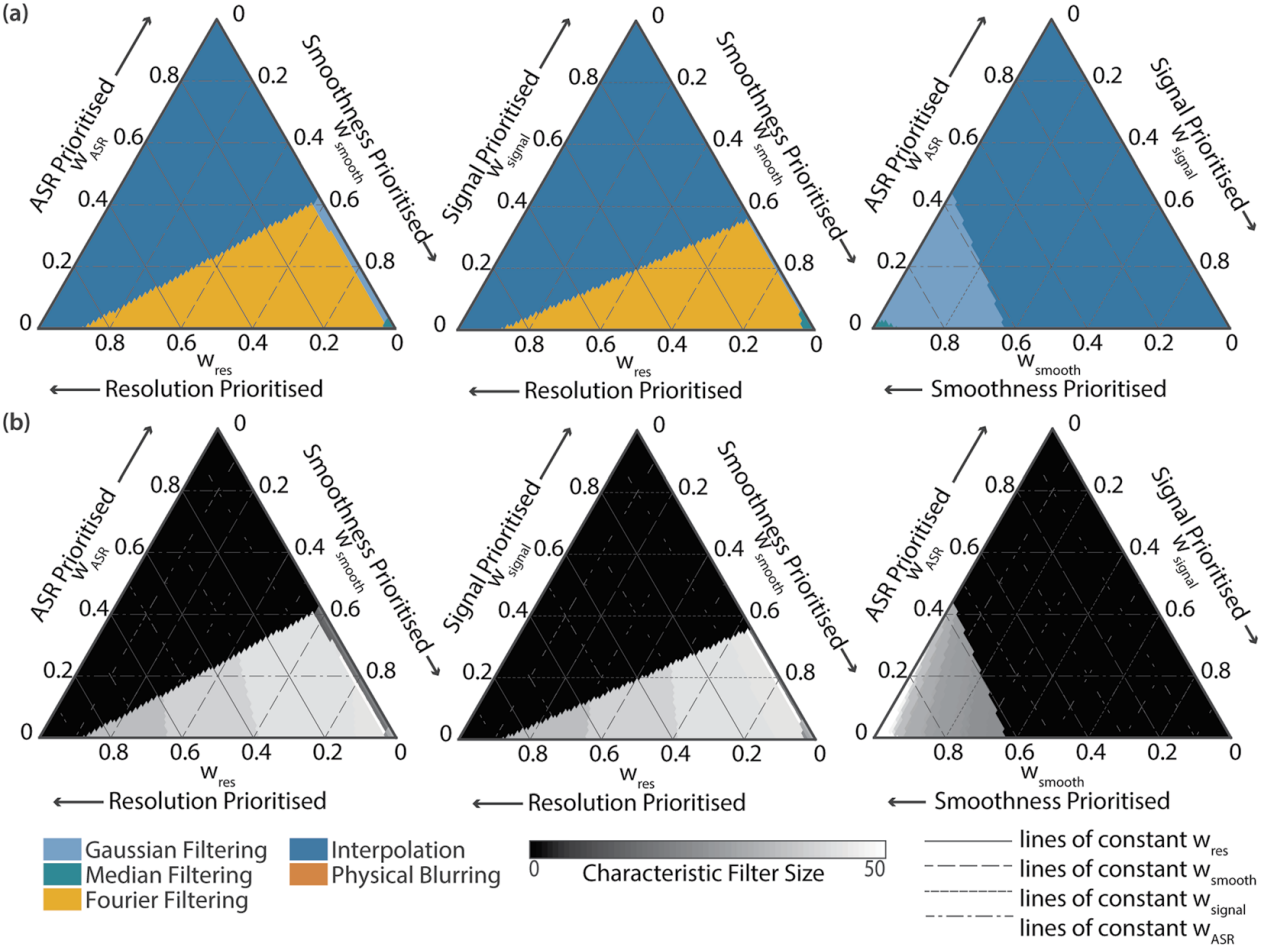
Optimum correction method based on overall performance score (OP) for weightings w_res_, w_smooth_, w_signal_ and w_ASR_. (**a**) The correction method that gives highest OP. (**b**) The characteristic filter size used with this correction method to achieve the highest OP. For each ternary plot, the weighting not shown on the axes is set to zero. Since interpolation between irregularly spaced points is a complex spatially variant filter, the characteristic filter size is not well defined, so it is represented as zero in the graphs. Speed is not included in the OP since it is possible to optimise speed independently. We do not show the trade off between resolution, smoothness and ASR, since in all combinations interpolation gives the best overall performance. Gaussian filtering (sky blue), median filtering (bluish green), Fourier filtering (orange), physical blurring (vermillion), interpolation (blue).

The overall performance is shown in Fig. 6. In the context of multispectral imaging, we find that interpolation frequently provides the optimal solution for correction, as was the case for monochrome imaging. Only if we prioritise smoothness does interpolation fail to provide the best solution. In this special case, Gaussian, median or Fourier filtering would be preferred, with Fourier filtering providing a better solution if we also wish to preserve resolution. Example multispectral images of colour scenes are shown in Supplementary Fig. 2.

## Discussion

Multispectral endoscopic imaging is an emerging fibrescope application in medicine. When implemented with spectrally resolved detector arrays (from simple 2 × 2 Bayer up to larger 3 × 3 + filter arrays) complexities arise in image processing for decombing and demosaicking. We sought to compare commonly used methods of decombing and evaluate their performance first in monochrome imaging and then establish how the performance would be affected by multispectral imaging using a spectrally resolved detector array. By defining scores relating to resolution, smoothness, signal, speed and accuracy of spectral reconstruction, we were able to test each of these correction methods within the parameter space of relevance for fibrescope imaging. We found that interpolation provides the optimal solution in most cases for both monochrome and multispectral imaging, failing only when image smoothness is highly prioritised, in which case Gaussian filtering is preferred. Fourier-filtering can be used in cases requiring a compromise between smoothness and resolution.

The optimal choice of correction method is application-dependent, hence rather than providing absolute recommendations, we objectively prepared overall performance scores composed of weighted sums of performance metrics. Weightings depend on the imaging priorities in a given application, which will vary significantly. To give some examples, visual inspection in real time puts speed as a high priority while inspection of detailed surface features, such as mucosal patterns or vasculature, will put resolution as a high priority. For autofluorescence imaging or fluorescence molecular imaging, where signal is relatively limited due to low abundance of fluorescence molecules *in vivo*, signal should be maximised to facilitate detection of these fluorescence markers. In cases requiring supervised classification of spectral signatures, such as in evaluation of oxy- and deoxy-haemoglobin concentrations during multispectral imaging, the accuracy of the spectral reconstruction is vital.

While previous works have established decombing methods for monochrome imaging, only interpolation has been used in multispectral imaging^47^. This previous method of implementation demosaicked the multispectral images prior to interpolation, which has several disadvantages. In particular, the fibre-centre-finding algorithm must be applied to each of the *L*^*2*^ individual spectral images (where *L* is the side length of a square super-pixel), requiring *L*^*2*^ manually input thresholds, and potential for spectral corruption is not well accounted for. In our work, we perform simultaneous demosaicking and decombing using a single fibre centre map generated from the *L*-fold higher resolution raw image. Furthermore, we explore four alternative correction methods for SRDA based multispectral fibrescope images, and assess them with respect to 5 performance metrics relevant to biomedical imaging applications.

Our results enable comparison of the performance of these different correction methods across a range of applications. Nonetheless, there remain some limitations to this study. Firstly, we only considered the case where the fibrelet image size on the sensor is greater than the size of a super-pixel. In practical applications, appropriate lenses could be used to magnify the image to ensure that this constraint is fulfilled. A consequence of this constraint is that demosaicking should not affect resolution. Indeed, the endoscopic imaging performed using the monochrome sensor and multispectral sensors had respective maximum resolutions of 240 ± 20 μm and 228 ± 18 μm (errors determined from errors in a linear fit to Michelson contrast data). Secondly, the magnification approach we used in simulation does not quite recapitulate all possible combs, since it magnifies both the cores and the cladding, whereas in reality, the same amount of cladding may be used or it may scale differently. Edge enhancing demosaicking methods^48^ were not investigated here, but could be applied to simultaneous decombing and demosaicking in future work. Finally, we did not consider the effect of compound methods such as interpolation followed by Gaussian smoothing.

## Conclusions

We assessed five comb correction methods with respect to five performance metrics relevant to biomedical imaging applications: the processing time, the resolution, the smoothness, the signal and the accuracy of spectral reconstruction. Interpolation provides the highest performance, failing only when image smoothness is highly prioritised, in which case Gaussian filtering is preferred. Otherwise, Fourier-filtering provides a good compromise between smoothness and resolution. With given weightings to each metric, which are determined by the particular imaging application, our results can be used to guide the selection of the correction method that best preserves overall performance.

## Methods

### Experimental system

The system is based around the PolyScope endoscope with disposable catheter sheath (PolyDiagnost, Germany), which can be introduced into the accessory channel of a larger endoscope (Fig. 1a). A narrow band ultra-high power LED (UHP-T-LED-630, Prizmatix, Israel) was coupled into the PolyScope illumination channel using a custom coupler (Prizmatix, Israel). The detection pathway consisted of an objective lens (NA = 0.5, UPLFLN20x, Olympus, Japan) and a tube lens (*f* = 100 mm, ACA254-100-A, Thorlabs, Germany), which focused light from the 10,000-fibrelet fibrescope onto a monochrome CMOS sensor (CMOSIS CMV4000-3E5) packaged into a USB3 camera (Grasshopper 3 GS3-U3-41C6M-C, Point Grey, Canada) or a monochrome CMOS sensor (NIR Ruby sensor, UI1242LE-NIR, IDS) packaged into a USB3 compact SRDA (CMS-V, SILIOS, France) (square pixel sizes 5.5 μm and 5.3 μm respectively). The SRDA consists of 9 spectral filters (8 narrow bands; average FWHM 30 nm; centre wavelengths 553, 587, 629, 665, 714, 749, 791, 829 nm; 1 broad band; 500–850 nm), which can be customized, deposited as a 3 × 3 super-pixels across the CMOS sensor. The spatial resolution of the endoscope is fundamentally limited by the Nyquist sampling limit at the plane of the imaging fibre tip, which is half the frequency of the fibrelet spacing – objects with higher spatial frequencies than this will experience aliasing. The spatial frequency limitation corresponds to different spatial resolutions at different working distances because the imaging optics do not project orthographically onto the fibre i.e. are not telecentric. The experimental system was configured such that at least one super-pixel fits within the image of a single fibrelet as recorded by the SRDA, ensuring each fibrelet captures complete spectral information.

### Simulations

We simulated monochrome images (outlined in Supplementary Fig. 3) using Matlab® 2016b (MathWorks, USA). Briefly, an experimental monochrome comb image was binarised to yield an ‘ideal’ comb mask; the mask was then magnified by different factors, M, relative to the original in order to investigate the effect of using different fibrelet diameters. We then generated a set of ‘ideal’ test images: a series of USAF targets to test resolution; an image with uniform intensity to test smoothness; and an image with a region of high intensity to test signal. The ideal binary comb masks and the test images were Gaussian blurred to represent imperfections in the fibrelets and imperfections in the test targets respectively. Noise was added to the ideal test images.

Next, each of the comb masks, $C(x,y)$, was used to mask each test image as follows:

1. The comb mask, $C(x,y)$, was split into individual fibrelet masks, ${C}_{i}(x,y)$.
2. Each individual fibrelet mask was binarised to generate binarised individual comb masks:
  $${C}_{i}^{B}(x,y)=\{\begin{array}{c}1,\,{C}_{i}(x,y) > 0.1\\ 0,\,{C}_{i}(x,y)\le 0.1\end{array}$$
3. In the region defined by ${C}_{i}^{B}(x,y),\,\,$the mean of the test image, $T(x,y)$, was taken:
  $${M}_{i}=\sum _{x,y}T(x,y){C}_{i}^{B}(x,y)/\sum _{x,y}{C}_{i}^{B}(x,y)$$
4. This was multiplied by the individual fibrelet masks, ${C}_{i}(x,y)$, to generate the simulated image, ${I}^{sim}$:

$${I}^{sim}(x,y)=\sum _{i}{C}_{i}(x,y){M}_{i}$$

Finally, Gaussian noise was added to reach the final simulated images.

### Monochrome image corrections

All image analysis was performed in Matlab® 2016b (MathWorks, USA). Five different decombing methods were applied directly to simulated and captured monochrome images. For all methods we define the amount of filtering by a dimensionless characteristic filter size r, which we varied. *Gaussian blur* was achieved by convolving the images with a 2-D Gaussian smoothing kernel with a standard deviation of *r* (1 < *r* < 50 pixels) via the Matlab function ‘imgaussfilt’. *Median filtering* was achieved by taking the median value of each 2r-by-2r-pixel region (1 < *r* < 50 pixels) via the Matlab the function ‘medfilt2’. For *Fourier filtering* a discrete Fourier transform of the image was performed via the Matlab fast Fourier transform function ‘fft2’. The Fourier transform image was cropped to remove any frequencies above *f*_0_, the cut-off frequency, and the inverse discrete Fourier transform applied using the Matlab function ‘ifft2’, to obtain the corrected image. The cut-off frequency was defined as:

$${f}_{0}\,=\,\frac{1}{2r}$$

where *r* is the characteristic filter size input (1 < *r* < 50 pixels) and corresponds to the smallest resolvable feature size in the inverse Fourier transformed image according to Nyquist’s theorem.

Interpolation relies on the location of each individual fibrelet in the image, for which we used an algorithm based on the work of Elter *et al*.^42^ similar to that applied in our previous work^49^:

1. Acquire a bright field calibration image *I*.
2. Candidate points are selected based on their brightness in relation to their local neighbourhood. Around each pixel *(x*,*y)* define a neighbourhood
  $$N=I(x-d:x+d,\,y-d:y+d)$$
  where the size of the neighbourhood *D* = *2d* + *1* and *D* is roughly the diameter of a single fibrelet.
  Given a minimal intensity difference *I*_*min*_, which is chosen by the user as the expected minimum intensity difference between fibrelets and cladding, a pixel *(x*, *y)* is considered as a candidate centre point if
  $$\max (N)-\,{\rm{\min }}(N) > {I}_{min}$$
3. For each candidate centre point, *(x*_*c*_, *y*_*c*_*)*, a score, *s*_*c*_, is calculated indicating how well a 2D quasi-Gaussian surface fits the neighbourhood around this point:
  $${s}_{c}=\sum _{N}{(G(x,y)-I(x,y))}^{2}$$
  where
  $$G=H{e}^{-{l}^{4}/2{D}^{2}}$$
  with *l* the distance from the candidate point and *H* = *I(x*_*c*_, *y*_*c*_*)*.
4. Order candidate centre points (*x*_*c*_, *y*_*c*_) by ascending score *s*_*c*_.
5. Starting from the highest ranked centre (lowest score), sequentially place each candidate fibre centre *(x*_*c*_, *y*_*c*_) onto a centre map *if and only if* the candidate centre is a minimum distance of one fibre diameter from all centres *(x*_*m*_, *y*_*m*_) already in the map:
  $$\sqrt{{({x}_{c}-{x}_{m})}^{2}+{({y}_{c}-{y}_{m})}^{2}} > D,\forall \,m$$
6. Fibre centres are added for as long as this criterion is satisfied until all candidates have been added to the map or rejected.

Decombing is then achieved using bilinear interpolation of the pixel values recorded at the fibre centres, *I(x*_*m*_, *y*_*m*_). We also performed physical blurring of our image by experimentally defocusing the image of the fibrescope face. We displaced the fibrescope along the optical axis by 5 μm in both directions and recorded images. In order to plot the results of physical blurring alongside the results of other correction methods, we arbitrarily defined the dimensionless characteristic filter size, *r*, as

$${\rm{r}}=\,|\frac{{\rm{displacement}}}{0.5\,\mu {\rm{m}}}|$$

### Multispectral image corrections

**Figure 7:**
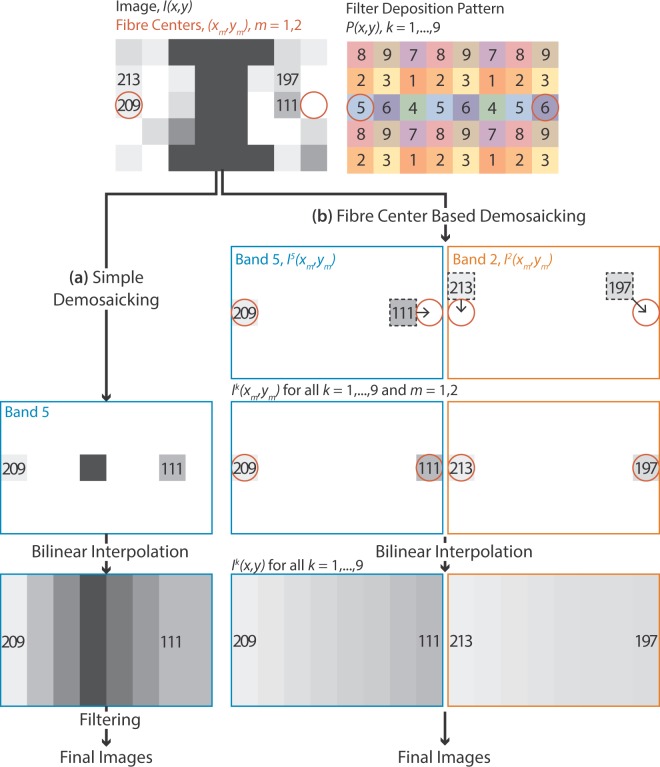
Schematic of the multispectral demosaicking and comb removal algorithms. An example image is shown with two fibrelet centres found as described in ‘Monochrome image corrections’. The corresponding filter deposition pattern for a 9-band colour filter array is shown to its right. (**a**) Simple demosaicking according to the filter deposition pattern is shown for band 5. Following this, bilinear interpolation is used to fill out the image. Finally, filtering of each image occurs, with either a Gaussian, median, or Fourier filter as described in ‘Monochrome image corrections’, or no filter in the case where physical blurring was applied to the image at the point of capture. (**b**) Fibre centre-based mosaicking is shown for bands 5 and 2 to illustrate the process outlined in detail in ‘Multispectral image corrections’. Briefly, at each fibre centre location, the intensity in each band is determined by taking the intensity at the nearest neighbouring pixel with the corresponding band filter. This is followed by interpolation between fibre centres, which can be implemented with a single look up table, since in every band and every image we interpolate between the same centre points.

Multispectral images consist of a mosaic of spectral information due to the filter deposition pattern of the CFA. In a process known as demosaicking (Fig. 7a), the final colour image is reconstructed by splitting the raw camera output into 9 incomplete mosaic pattern images that are subsequently interpolated. Demosaicking must occur prior to decombing with Gaussian, median and Fourier filtering, as mixing information from adjacent pixels on the raw image would corrupt the recorded spectral information. When physical blurring was applied to the image at the point of capture, light spreads from each fibrelet decombing the image prior to its passage through the CFA, so following demosaicking, no further filtering takes place.

Interpolation between fibrelet centres must occur in parallel to demosaicking (Fig. 7b). This was implemented as follows:

1. Acquire a bright field calibration image, *I(x*, *y*), with broadband illumination to ensure a signal is recorded in all spectral bands.
2. Apply a Gaussian blur to the bright field calibration image in order to smooth out the mosaic pattern due to the CFA.
3. Find the centres of the fibrelets, *(x*_*m*_, *y*_*m*_), using steps 2–6 of the centre finding algorithm outlined in ‘Monochrome image corrections’.
4. At each fibrelet centre point *(x*_*m*_, *y*_*m*_), we need to know the image intensity in each spectral band *k*, *I*^*k*^(*x*_*m*_, *y*_*m*_). The centre point corresponds to a pixel with one spectral filter, giving us the intensity in one of the spectral bands. For the other spectral bands, we assume the intensity at the centre point is the same as the intensity at the nearest neighbour with the correct spectral filter. We know the filter deposition pattern on the sensor:

$$P(x,y)=\,\{\begin{array}{c}1\\ 2\\ \vdots \end{array}$$

So we can say:

$${I}^{k}({x}_{m},{y}_{m})=I({x}_{NN},{y}_{NN})$$

where *(x*_*NN*_, *y*_*NN*_*)* is the nearest neighbour pixel with the spectral filter *k*:

$$P({x}_{NN},{y}_{NN})=\,k$$

This process should result in image data for all spectral bands and at all centre locations:

$${I}^{k}({x}_{m},{y}_{m})\forall k,m$$

This is generalisable to any size mosaic as long as the ratio, *T*, between the super-pixel size and the fibrelet size on the sensor, obeys the criterion:

$$T=\frac{L}{D} < 1$$

where *L* is the side length of a super-pixel and *D* is the diameter of the fibrelets on the sensor. Though not used here, even valued super-pixels (e.g. 2 × 2, 4 × 4) can result in some image points having two equally distant nearest neighbours in some spectral bands so an appropriate randomised selection or average of these would need to be taken. Finally, we reconstruct a comb free image for each spectral band using bilinear interpolation of the pixel values at fibre centres within that spectral band.

### Performance metrics

In order to determine the performance of comb removal, we assessed 4 performance metrics for monochrome imaging and 5 performance metrics for multispectral imaging.

#### Resolution

Resolution was determined by capturing images of a 1951 USAF resolution test target (#53–714, Edmund Optics, USA) illuminated externally with a broadband halogen light source (OSL2, Thorlabs, Germany). The Michelson contrast^50^ was calculated for each element. The resolution, R, was determined as the line spacing when Michelson contrast dropped below 5% by fitting a smoothing spline to the Michelson contrast versus the line spacing. A contrast threshold of 1% has previously been reported to be applicable across a wide range of targets and conditions^50^, but we chose 5% to avoid effects arising from noise at very low contrast. We also placed a condition requiring data points with contrast >1% at >3 distinct line spacings to ensure there were a reasonable number of non-noise data points to fit a spline. If this condition was not met, a spline was not fitted and the resolution was not defined. For multispectral imaging, the resolution is taken as the average of the resolution determined for each of the 9 spectral bands. The resolution score^39^, *S*_*res*_, was defined as

$${S}_{res}=1-(\frac{R-{R}_{min}}{{R}_{max}-{R}_{min}})$$

where *R*_*max*_ and *R*_*min*_ are the maximum and minimum resolutions calculated across all correction methods, defined such that scores of 1 and 0 represent the best and worst resolution achieved respectively.

#### Smoothness

For monochrome imaging, smoothness was determined by using images of white areas of a 1951 USAF resolution test target (#53–714, Edmund Optics, USA) illuminated externally with a broadband halogen light source (OSL2, Thorlabs, Germany). For multispectral imaging, smoothness was determined using images of a white reflecting target (paper) illuminated with a narrow band LED (UHP-T-LED-630, Prizmatix, Israel).

The spatial standard deviation of the image was calculated^39^. The average of this across 9 spectral band images was taken for multispectral imaging. The smoothness score^39^, *S*_*smooth*_, was defined as:

$${S}_{smooth}=1-(\frac{\sigma -{\sigma }_{min}}{{\sigma }_{max}-{\sigma }_{min}})$$

where *σ*_*max*_ and *σ*_*min*_ are the maximum and minimum standard deviations calculated across all correction methods, such that scores of 1 and 0 represent the best and worst smoothness achieved respectively.

#### Signal

Fluorescence signals with broad spectral features were acquired by capturing images of a 30 μL solution of 1 mg/mL of the fluorescent dye AF647 (Invitrogen, USA) dissolved in phosphate buffered saline (PBS) in a well plate (μ-Slide 18 Well—Flat, ibidi GmbH, Germany) using illumination from a 630 nm LED (UHP-T-LED-630, Prizmatix, Israel) and a long pass emission filter (ET700/75 m, Chroma, USA). The mean pixel intensity, *S*, was calculated in a region of interest (ROI) drawn manually inside the well on the image. For multispectral imaging, the average pixel intensity was extracted from those bands that overlap with the emission spectrum of AF647 (narrow bands: 665 nm, 714 nm, FWHM, 27 nm, 26 nm; broadband: 500–850 nm).

The average pixel intensity, *S*, was used to determine the signal score *S*_*signal*_

$${S}_{signal}=\,\frac{S-{S}_{min}}{{S}_{max}-{S}_{min}}$$

where *S*_*max*_ and *S*_*min*_ are the maximum and minimum signals calculated across all correction methods, such that scores of 1 and 0 represent the best and worst signal achieved respectively.

#### Speed

Speed was determined by measuring the total Matlab® computation time per frame on a MacBook Pro (Processor 2.4 GHz Intel Core i5, Memory 8 GB 1600 MHz DDR3).

#### Accuracy of spectral reconstruction (Multispectral Performance Metric Only)

For multispectral imaging, it is crucial that the information from different pixels on the SRDA is not mixed by the comb correction process. In order to assess the performance of each correction method, we defined a score to represent the accuracy of spectral reconstruction (ASR). To extract this score, the following steps were performed:

1. Using our multispectral endoscope, we captured images of a white reflecting target (paper) illuminated with a narrow band source (UHP-T-LED-630, Prizmatix, Israel).
2. These images were demosaicked and decombed as outlined in Section 5.4 to provide an MSI cube of data:$\,{I}^{k},\,k=1-9$ where *k* indicates the spectral band.
3. The ‘ground truth’ spectral properties of the image data were determined. The spectrum, *G(λ)*, of the target was captured using a reference spectrometer (AvaSpec-ULS2048, Avantes, Netherlands); the spectral response of our endoscope in each spectral band *k*, *R*^*k*^*(λ)*, was determined as described previously^38^.
4. The ‘ground truth’ spectrum was multiplied by the response of our endoscope to predict the ‘ground truth’ recorded spectrum:

$${E}^{k}=\sum _{\lambda }G(\lambda ){R}^{k}(\lambda ),\,k=1-9$$

The normalised (to AUC = 1) average (over all pixels in the image) spectrum collected with the endoscope was compared to the predicted ‘ground truth’ spectrum and the mean squared difference was determined by:

$$Q=\sum _{k=1}^{9}{(\overline{(\frac{{\sum }_{x,y}{I}^{k}(x,y)}{{\sum }_{x,y}1})}-\overline{{E}^{k}})}^{2}$$

where the bar represents normalisation of spectra to AUC=1.

The accuracy of spectral reconstruction score, *S*_*ASR*_, is defined as

$${S}_{ASR}=\,1-(\frac{Q-{Q}_{min}}{{Q}_{max}-{Q}_{min}})$$

where *Q*_*max*_ and *Q*_*min*_ are the maximum and minimum mean squared differences calculated across all correction methods, such that scores of 1 and 0 represent the best and worst S_ASR_ achieved respectively.

By spatially (pixel-by-pixel) averaging the spectra prior to normalisation and calculation of the mean square difference, we reduce the influence of non-smoothness of the images, since this effect is already accounted for in the smoothness metric.

### Overall performance

Since there are trade-offs between the performances of the metrics, the overall performance of a particular correction method depends on which of the metrics are prioritised in a given application. To account for this, we constructed an overall performance score, *OP*, defined as:

$$OP={w}_{res}{S}_{res}+{w}_{smooth}{S}_{smooth}+{w}_{signal}{S}_{signal}+{w}_{ASR}{S}_{ASR}$$

with adjustable application-dependent weightings, *w*, to emphasize a priority metric, such that:

$${w}_{res}+{w}_{smooth}+{w}_{signal}+{w}_{ASR}=1$$

Speed was not included in *OP* since it is possible to independently optimise speed by improving hardware and parallelising software. For monochrome imaging *w*_*ASR*_ = 0. Since weightings are application-dependent, we calculated *OP* for all weightings, such that the reader may visually select the optimum correction method.

## Electronic supplementary material

Supplementary Information

## Data Availability

The datasets generated and analysed during the current study are available in the University of Cambridge Research Data Repository (DOI will be generated upon acceptance of manuscript).
