# Supplementary material for: Quantitative evaluation of comb-structure correction methods for multispectral fibrescopic imaging
Source: Sci Rep. 2018 Dec 12;8:17801. doi: 10.1038/s41598-018-36088-7 (PMC6290790; doi:10.1038/s41598-018-36088-7)
Supplement: Supplementary file 1 — Supplementary Information [file 41598_2018_36088_MOESM1_ESM.pdf]

Title

Quantitative evaluation of comb-structure correction methods for multispectral fibrescopic imaging

Authors

Dale J. Waterhouse[1,2], A. Siri Luthman[1,2], Jonghee Yoon[1,2], George S D Gordon[1,2,3] and Sarah E. Bohndiek[1,2*]

1. Department of Physics, University of Cambridge, CB3 0HE, UK
2. Cancer Research UK Cambridge Institute, University of Cambridge, CB2 0RE, UK
3. Department of Engineering, University of Cambridge, CB3 0FA

*e-mail: seb53@ cam.ac.uk

Supplementary Figures

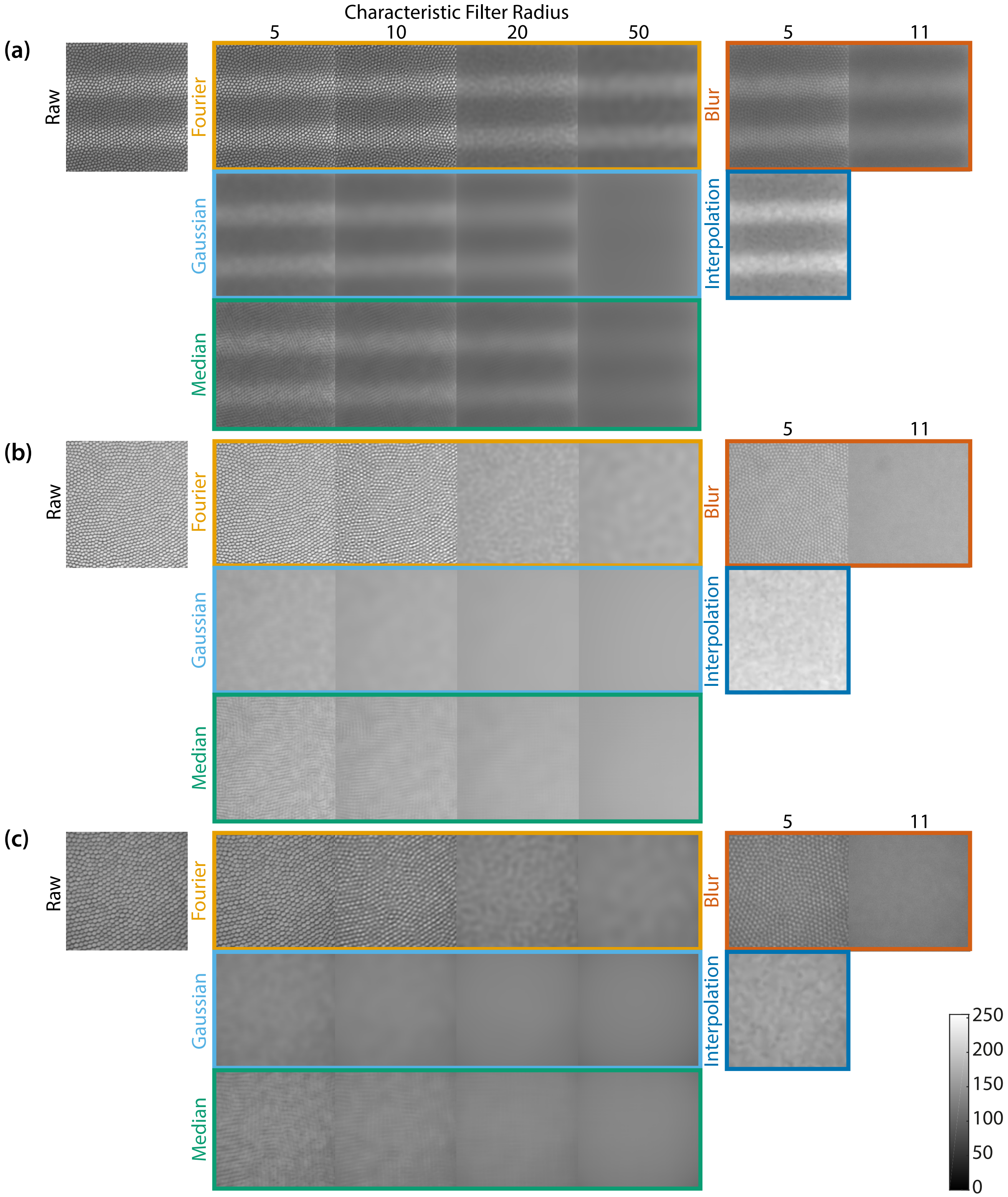

Supplementary Figure 1. Example corrected monochrome images. (a). Images of USAF test chart element used to determine resolution. **(b).** Images of white reflecting target (paper) used to determine smoothness. **(c).** Images of AF647 used to determine signal.

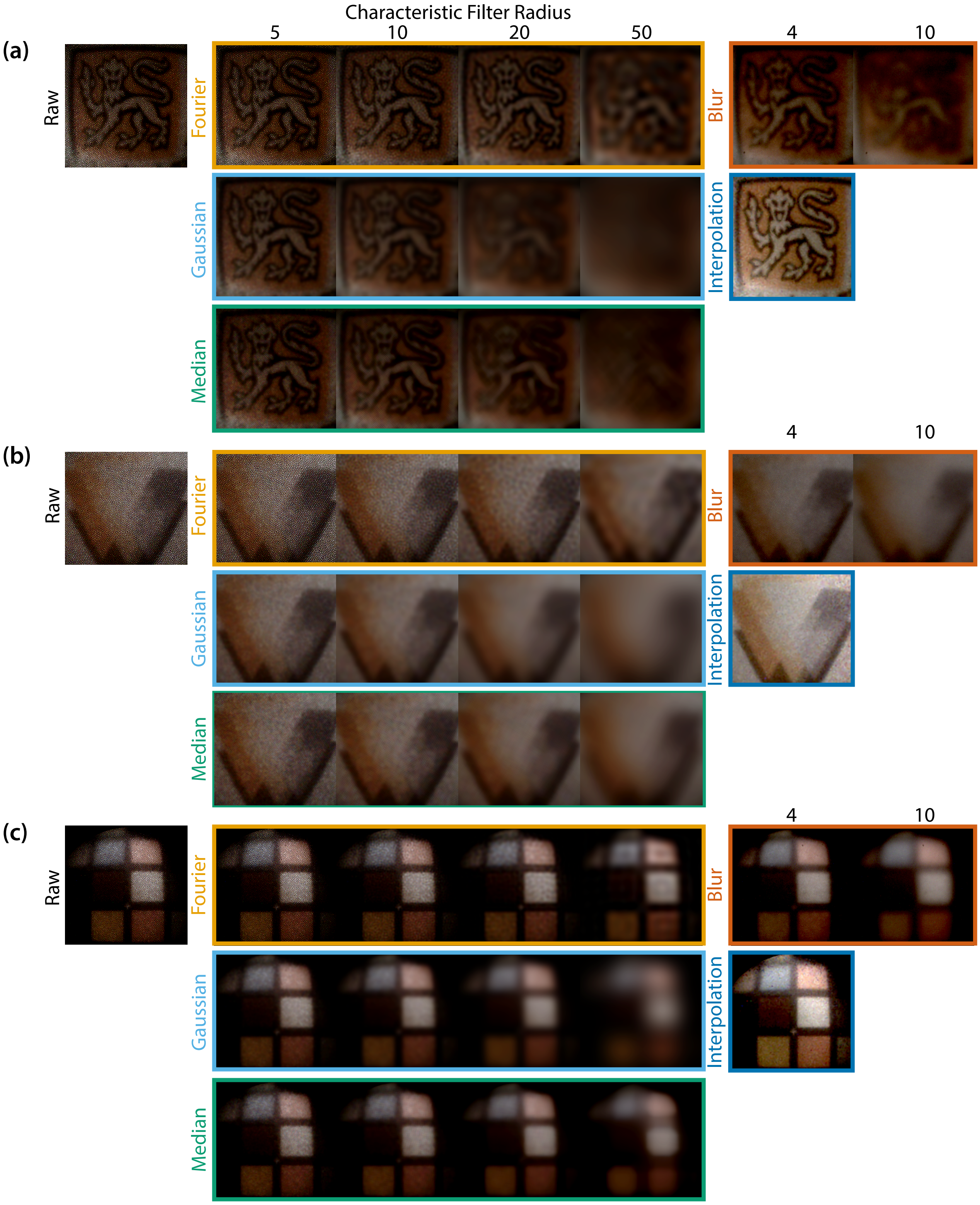

Supplementary Figure 2. Example corrected multispectral images. (a). Images of a printed University of Cambridge crest. **(b).** Images of a printed VISIONLab logo. **(c).** Images of a Macbeth ColorChecker (X-Rite, USA) (elements shown are left-right top-bottom: 11, 17, 10, 18, 9, 16). The false colour images were generated by assigning the narrow bands centred at 629, 587 and 553nm to RGB channels respectively.

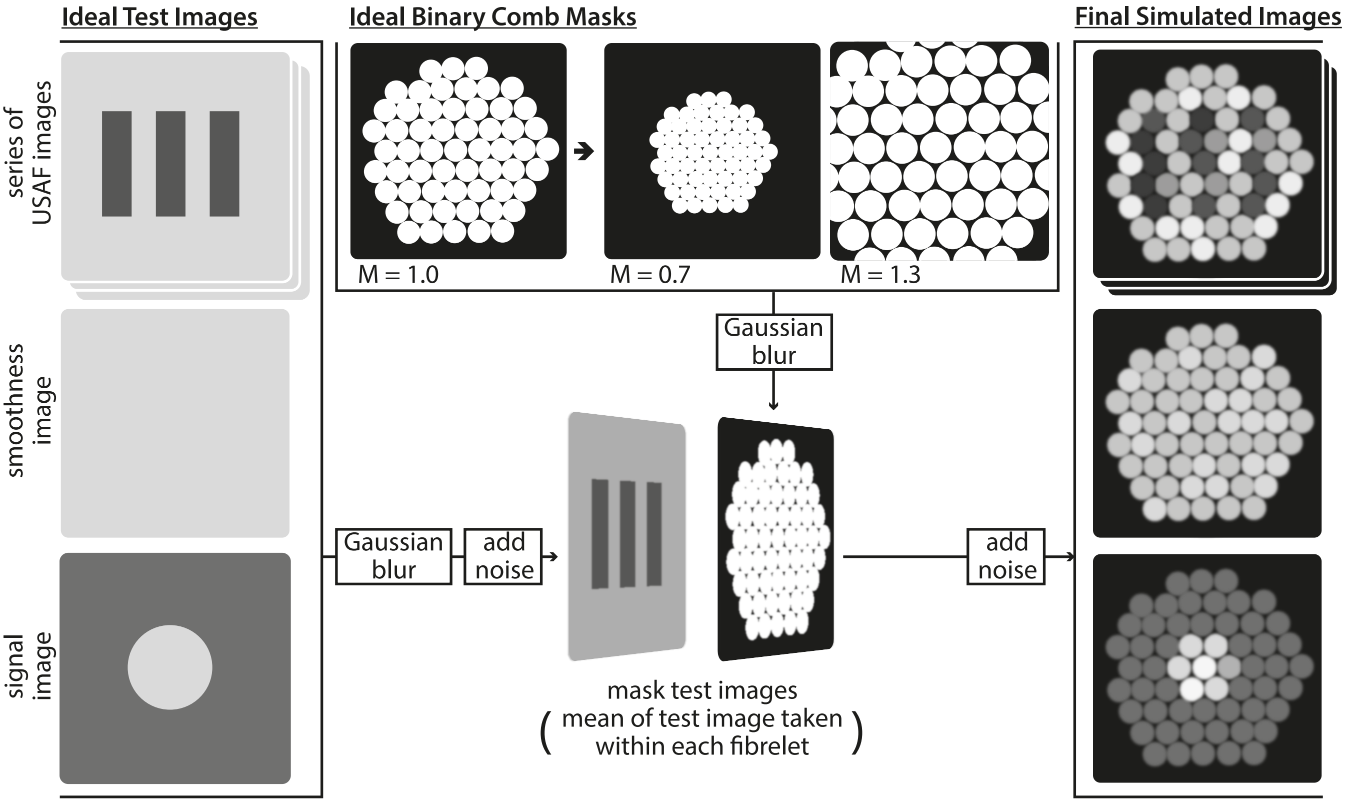

Supplementary Figure 3. Schematic of the image simulation process. An experimental comb image was binarised to yield an ‘ideal’ irregular binary comb mask. This was stretched by a factor, M, compared to the original to create stretched binary comb masks. ‘Ideal’ test images were generated; a series of USAF targets to test resolution, an image with uniform intensity to test smoothness and an image with a region of high intensity to test signal. The ideal binary comb masks and the test images were blurred to represent imperfections in the fibrelets and imperfections in the test targets respectively. Noise was added to the ideal test images. Next, each of the comb masks was used to mask each test image as follows. The comb mask was split into individual fibrelet masks and for each fibrelet the mean of the test image in the region where the intensity of the fibrelet mask was >0.1 was taken, then multiplied by the fibrelet mask, and added to the final image. Noise was added to reach the final simulated images.
